# Effect of Sphingomyelin and Vitamin D3 Intake on the Rabbit Brain

**DOI:** 10.3390/ijms26073269

**Published:** 2025-04-01

**Authors:** Federico Fiorani, Alda Quattrone, Samuela Cataldi, Roberto Maria Pellegrino, Carla Emiliani, Cataldo Arcuri, Alessandra Mirarchi, Giulio Curone, Laura Menchetti, Tommaso Beccari, Claudia Floridi, Martina Mandarano, Gabriele Brecchia, Elisabetta Albi

**Affiliations:** 1 Department of Pharmaceutical Sciences, University of Perugia, 06126 Perugia, Italy; federico.fiorani@dottorandi.unipg.it (F.F.); samuela.cataldi@gmail.com (S.C.); beccari.tommaso@unipg.it (T.B.); 2 Department of Veterinary Medicine, University of Milan, 26900 Lodi, Italy; alda.quattrone@unimi.it (A.Q.); giulio.curone@unimi.it (G.C.); gabriele.brecchia@unimi.it (G.B.); 3 Department of Chemistry, Biology and Biotechnology, University of Perugia, 06123 Perugia, Italy; roberto.pellegrino@unipg.it (R.M.P.); carla.emiliani@unipg.it (C.E.); 4 Department of Medicine and Surgery, University of Perugia, 06126 Perugia, Italy; cataldo.arcuri@unipg.it (C.A.); alessandra.mirarchi@dottorandi.unipg.it (A.M.); 5 School of Bioscience and Veterinary Medicine, University of Camerino, 62024 Matelica, Italy; laura.menchetti@unicam.it; 6 Section of Anatomic Pathology and Histology, Department of Medicine and Surgery, University of Perugia, 06126 Perugia, Italy; claudia.floridi@unipg.it (C.F.); martina.mandarano@unipg.it (M.M.)

**Keywords:** brain, diet, rabbit, sphingomyelin, vitamin D3

## Abstract

Sphingomyelin is a crucial molecule in the sphingolipid metabolic pathway, and its action is closely related to that of vitamin D3. Both molecules are recognized for their involvement in brain pathophysiology. In this study, the effect of a sphingomyelin + vitamin D3-enriched diet was investigated in rabbits. The results showed a strong immunopositive GFAP staining in the brain’s white matter. Furthermore, a remodeling of the balance between glycero-phospholipids and ether-phospholipids was observed in the brain, along with an increase in ceramides and hexose ceramides, molecules relevant for the structure, function, and stability of myelin. Taken together, these findings provide clues as to how the combination sphingomyelin + vitamin D3 may play a vital role in normal brain physiology and could potentially be leveraged in the context of neurodegenerative diseases.

## 1. Introduction

Sphingomyelin (SM) is recognized as the largest member of the sphingolipid (Sph) family, a class of lipids involved in diverse physiopathological processes, including cell proliferation, differentiation, apoptosis, and cancer [1]. The structural foundation of Sph molecules is sphingosine (SP), a rigid molecule containing both alcohol and amino groups [2] (Goñi, 2022). Among Sphs, SM exhibits several unique features: (1) it is synthesized de novo from serine, an amino acid, and palmitic acid, a fatty acid; (2) it can also be synthesized through an alternative pathway, in which phosphatidylcholine (PC) donates a phosphocholine group to ceramide (Cer), freeing diacylglycerol (DAG); (3) it is degraded by sphingomyelinase (SMase), yielding Cer; (4) it can bind cholesterol (Chol) via van der Waals forces to form membrane rafts; (5) it forms rafts with Chol in the nuclear membrane; and (6) it is a critical component of the myelin sheath.

These properties highlighted SM as a molecule with extensive metabolic interactions and a broad range of biological activities. Notably, the dual synthesis mechanisms of SM are significant. On one hand, de novo synthesis depends on serine and palmitic acid availability and occurs in the endoplasmic reticulum [3]. On the other hand, SM can be synthesized from phosphatidylcholine (PC) through SM-synthase [4]. Conversely, PC can be synthesized from SM through reverse SM-synthase activity [5]. Therefore, the PC-SM metabolic interplay regulates the pools of DAG and Cer, two second messengers with contrasting roles: DAG promotes cell proliferation, while Cer is driving differentiation and/or apoptosis [6]. In the cell nucleus, the PC-SM and DAG-Cer metabolic cycles act as regulatory clocks for gene expression [7].

Furthermore, the Cer produced from SM degradation might follow distinct pathways: (1) it can be hydrolyzed by ceramidase to sphingosine (SP), which can be phosphorylated to sphingosine-1-phosphate (S1P), a signaling molecule crucial in several physiopathological processes; (2) it can combine with sugars to form glucocerebrosides and gangliosides, essential components of the central nervous system. Thus, SM and its metabolites are considered indispensable for brain function [8]. Dysregulated SM metabolism has been implicated in depression, schizophrenia, Alzheimer’s disease (AD), Parkinson’s disease (PD), and Niemann–Pick disease (NPD) [1].

An interplay between SM and vitamin D3 (VD3) has been described. VD3 stimulates SMase activity, leading to SM degradation and Cer production at the cellular level [9]. At the same time, SM is essential for VD3 interaction with its nuclear receptor (VDR) [10]. The multifaceted roles of VD3 at cellular and nuclear levels have been described [11]. By influencing Cer production and its downstream metabolites, including S1P, glucocerebrosides, and gangliosides, VD3 is involved in brain physiology and neurodevelopment [10]. VD3 deficiency has been associated with neuropathic pain [12], traumatic brain injury [13], and neurodegeneration [14].

Moreover, SM interacts with Chol to form lipid rafts, which play distinct roles in cellular and nuclear membranes [10,15,16]. At the nuclear level, lipid rafts anchor active chromatin by influencing gene transcription. In neuronal cells, SM is critical for cell membrane integrity, myelin sheath formation, and VD3-mediated nuclear functions.

Both SM and VD3 are abundant in milk, dairy products, and egg yolk, as well as in fish, almond, pecans, pine nut, walnut, and microalgae, making them accessible through a balanced diet [17]. However, the complexity of food matrices complicates the precise determination of their individual roles. To date, the specific effects of SM and VD3 administered orally have not been fully explored. For the patenting of an SM-VD3 product, in vitro experiments were conducted on embryonic hippocampal cells of the HN9.10e line, in which SM, VD3, or the SM-VD3 combination were administered separately at different concentrations and times. Considering the metabolic interaction between SM and VD3 reported above, we could hypothesize that a dietary supplementation with an SM-VD3 combination could influence brain metabolism. Thus, the aim of this study was to verify the effect of dietary supplementation with a patented formulation SM-VD3 in the brain of rabbits after 4 months of treatment, verifying the possible influence on monthly plasma PLs and on intestinal PLs after 4 months of treatment.

## 2. Results

### 2.1. SM-VD3 Does Not Influence the Growth of Rabbits

**Figure 1:**
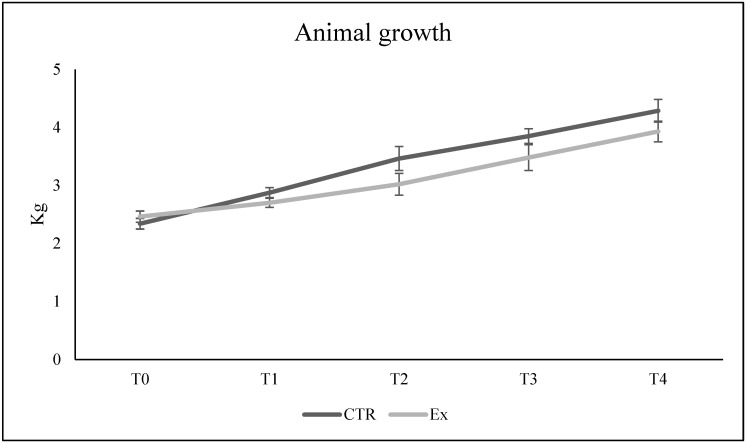
Growth curve of rabbits fed a regular diet enriched with a daily dose of a formulation consisting of SM-rich phospholipids + VD3 for 4 months. Weight was checked every month, at T0 (time 0) and after 1, 2, 3 and 4 months of treatment, corresponding to T1, T2, T3, and T4. The data represent the average weight ± SD of 6 control animals (CTR) and 6 experimental animals (Ex). Student’s *t*-test was used to analyze the significance of the results in Ex animals versus CTR animals, but no differences between CTR and Ex were statistically significant.

We first sought to verify that the daily administration of SM-VD3 did not influence the progressive growth of the rabbits over time. As shown in Figure 1, at the beginning of the experiment, the rabbits had a weight of about 2.3–2.5 kg (physiological for their age, corresponding to 2 months of life). After 2, 3, and 4 months, the growth curve showed a similar trend in CTR and Ex animals. In the Ex rabbits, the curve was slightly lower, although the differences compared to CRT were not statistically significant (Supplementary Table S1).

### 2.2. SM-VD3 Induces Changes in Plasma Phospholipids

**Figure 2:**
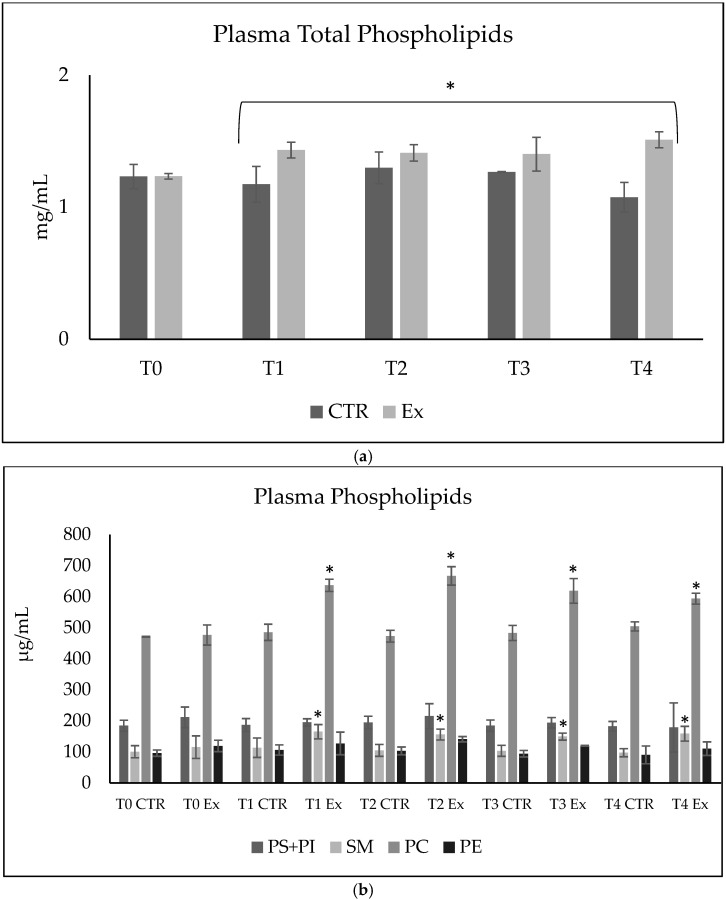
Total phospholipids (**a**) and each class of phospholipids (**b**) in plasma from rabbits that were untreated (CTR) or treated with a daily dose of SM-VD3(Ex). Blood from 6 CTR and 6 Ex animals was taken at 0 time and after 1, 2, 3, and 4 months of treatment, corresponding to T0, T1, T2, T3, and T4. The data represent the mean ± SD for 6 CTR and 6 Ex animals. Phosphatidylserine (PS), phosphatidylinositol (PI), sphingomyelin (SM), phosphatidylcholine (PC), phosphatidylethanolamine (PE). Significance was analyzed by *t*-test; * *p* < 0.05 versus CTR.

In order to study the role of SM-VD3 formulation in regulating plasma PL levels, we first chose to analyze the level of total PLs. The results showed that the plasma PL level in the Ex animals tended to increase slightly compared to that in the CTR animals, already after 1 month of treatment. The trend continued up to 4 months (Figure 2a, Supplementary Table S2).

We were further interested in the changes in the main classes of PLs with the diet due to previous studies demonstrating diet-induced changes in blood lipid profiles [18]. Thus, we investigated the possible change in the total content of phosphatidylserine (PS) + phosphatidylinositol (PI), SM, phosphatidylcholine (PC), and phosphatidylethanolamine (PE). As expected, PC was the most abundant lipid, followed by PS + PI, SM, and PE. In the CTR samples, plasma PL composition remained constant over time. In the Ex samples, an increase in SM and PC was observed at T1, T2, T3, and T4 compared to the respective CTR samples (Figure 2b, Supplementary Table S3).

### 2.3. Effect of SM-VD3 on Intestine

**Figure 3:**
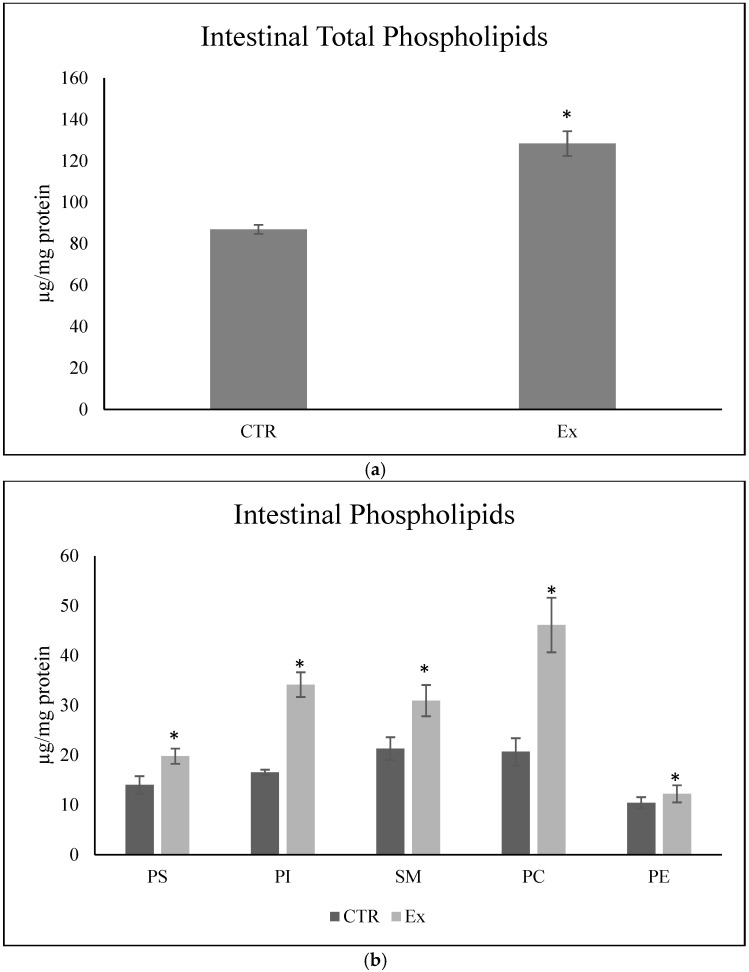
Total phospholipids (**a**) and each class of phospholipids (**b**) in the intestines from rabbits untreated (CTR) or treated with a daily doses of SM-VD3(Ex). Animals were sacrificed and the intestine was removed after 4 months of treatment, 1 h after the daily dose of SM-VD3. The data represent the mean ± SD of 6 CTR and 6 Ex animals. Significance was analyzed by *t*-test; * *p* < 0.05 versus CTR.

The variations in plasma PL levels could be attributed to increased intestinal absorption and/or to their metabolism at tissue level. Thus, we wanted to investigate whether an increase in PLs was actually found in the intestine of animals that had received the daily dose of SM-VD3 1 h before sacrifice. All the PLs investigated were increased in the Ex animals compared to the CTR animals (Figure 3, Supplementary Table S4).

**Figure 4:**
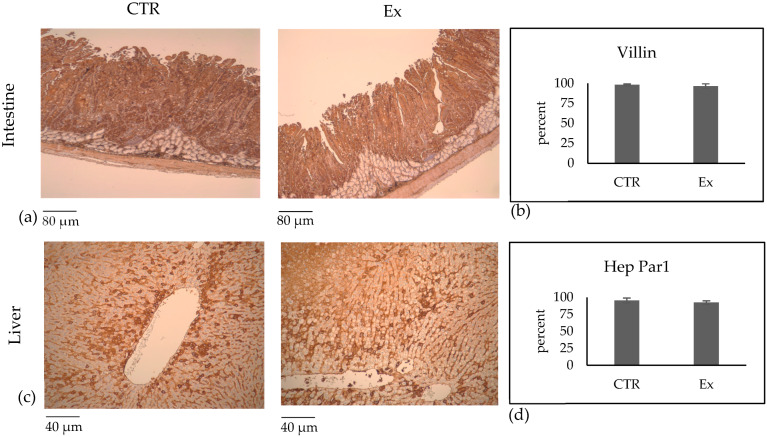
Immunohistochemical analysis of villin as marker for intestine cell differentiation and Hep Par1 for hepatocellular cell differentiation. The animals were sacrificed after 4 months of treatment with SM-VD3, 1 h after the daily dose. The liver and the intestine were removed. CTR, control animal; Ex, experimental animal. The analysis was performed, and images were acquired, as reported in Section 4. For the intestine, images were acquired at 50× magnification (**a**); for the liver, images were acquired at 100× magnification (**c**). (**b**,**d**) Evaluation of the percentage of positive cells. The data represent the mean ± SD of 10 different areas from 4 sections for each animal. Student’s *t*-test was used to analyze the significance of the results for the Ex animals versus the CTR animals, but no differences between the CTR and Ex animals were significant.

To ensure that SM-VD3 did not induce alterations in the intestinal epithelium, we decided to evaluate the structure of the intestinal wall and the expression of villin, a marker of epithelial cell differentiation [19]. The results showed that the structure of the intestine was perfectly preserved (Figure 4a). The same figure shows immunohistochemistry analysis performed with a specific anti-villin antibody. The stain intensity was classified as 0 = absent; 1 = weak; 2 = intermediate; 3 = strong. The villin staining was classified as level 3. The percentage of cells showing level 3 labeling was calculated and is presented in the graph. The results showed that the percentage of labeling was 98.1 ± 1.2 and 96.4 ± 3.0 in the intestines of the CTR and Ex animals, respectively (Figure 4b).

We next asked whether SM-VD3 could impact hepatocyte, given the liver’s role as a lipid-metabolizing organ. Thus, we checked the structure of liver tissue and the expression of Hep Par 1, a marker of hepatocellular differentiation [20]. The structure was completely preserved (Figure 4c). The Hep Par 1 staining was classified as level 1. The percentage of cells with level 1 labeling was calculated and is presented in the graph (Figure 4d). The results showed that the percentage of labeling was 95.5 ± 3.5 and 92.5 ± 2.5 in the livers of the CTR and Ex animals, respectively (Figure 4d).

### 2.4. Effect of SM-VD3 on the Brain

SM and VD3 were considered essential molecules for brain function [9,11,13,14,15]. Thus, we studied GFAP expression by immunohistochemistry and immunofluorescence in rabbit brains supplemented with SM-VD3 formulation in the daily diet and compared the results with those obtained in the brains of the rabbits that were not supplemented with the formulation in the daily diet.

Our results, obtained after 4 months of daily administration of SM-VD3, showed variations in astrocytic glial acidic fibrillary protein (GFAP) expression, an intermediate filament protein expressed predominantly by astrocytes [21]. Of note, the stain intensity was classified as follows: 0 = absent; 1 = weak; 2 = intermediate; 3 = strong. The GFAP staining was classified as level 3. The percentage of cells with level 3 labeling was calculated and is presented in the graph.

**Figure 5:**
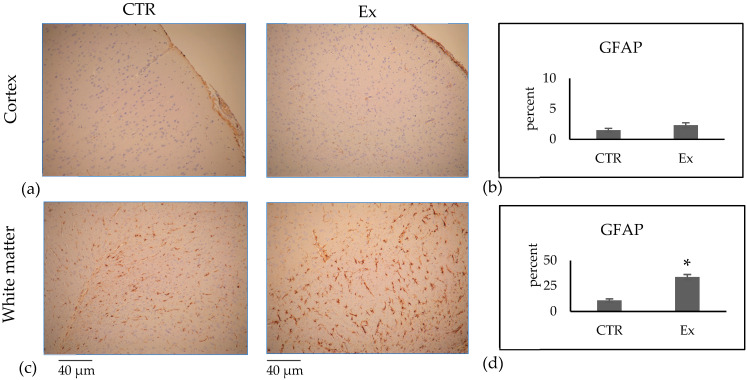
Immunohistochemical analysis of GFAP as astrocyte marker. Animals were sacrificed after 4 months of treatment with SM-VD3, 1 h after the daily dose. The brain was removed and the cortex or white matter was isolated, as reported in Section 4. CTR, control animals; Ex, experimental animals. The analysis was performed, and the images were acquired as reported in Section 4. Images were acquired at 100× magnification (**a**,**c**). (**b**,**d**) Evaluation of the percentage of positive cells. The system automatically evaluates 10 areas of each slice. The values from the 4 sections of each slice were combined to provide one data point for each animal. Data represent the mean ± SD for n° 6 CTR and n° 6 Ex animals. Therefore, the *t* test probability was calculated with the data obtained from each animal; * *p* < 0.05 versus CTR.

The results showed that the percentage of labeling was 1.5 ± 0.3 and 2.3 ± 0.4 in the cortex of the control (CTR) and experimental (Ex) groups, respectively, and 10.7 ± 1.6 and 33.8 ± 2.5 in the white matter of the CTR and Ex groups, respectively, indicating a 3.1-fold increase in GFAP expression only in the white matter (Figure 5c,d).

**Figure 6:**
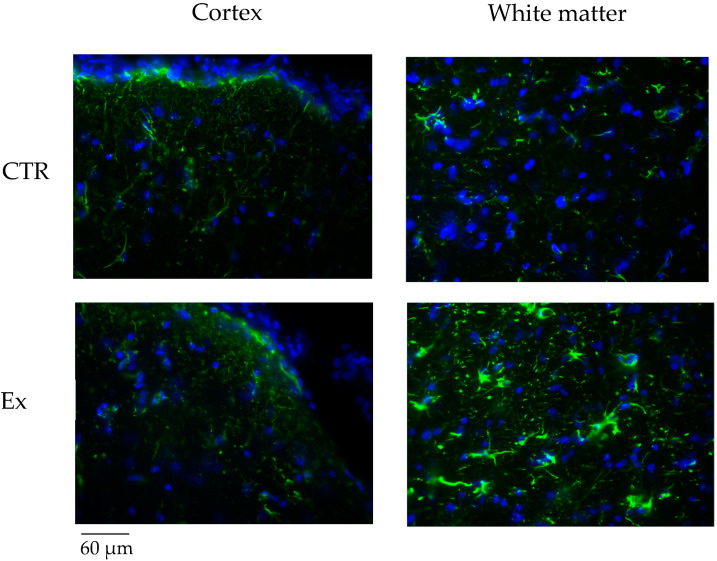
Immunofluorescence analysis of GFAP. Animals were sacrificed after 4 months of treatment with SM-VD3, 1 h after the daily dose. The brain was removed and the cortex or white matter was isolated as reported in Section 4. CTR, control animal; Ex, experimental animal. GFAP is shown in green; nuclei are shown in blue. The analysis was performed and images were acquired as reported in the Section 4. Images were acquired at 40× magnification.

Immunofluorescence analysis confirmed these data (Figure 6).

In order to test whether brain PLs changed after 4 months of SM-VD3 daily administration, we performed lipidomic analysis on a pool of lipid extracted from the CTR samples and a pool of lipid extracted from the Ex samples. Therefore, we grouped together all the species within each individual PL family: glycerophospholipids (GPLs), including phosphatidylethanolamine (PE), phosphatidylcholine (PC), phosphatidylinositol (PI), phosphatidylserine (PS); ether GPLs (GPLs O), including ether PE (PE O), ether PC (PC O), ether PI (PI O), ether PS (PS O); lyso GPLs (LGPLs), including lyso PE (LPE), lyso PC (LPC), lyso PI (LPI), lyso PS (LPS); ether lyso GPLs (LGPLs O), including ether LPE (LPE O), ether LPC (LPC O); phosphatidylglycerol (PG) and lyso PG (LPG); sphingolipids (Sphs), including sphingomyelin (SM), ceramide (Cer), hexosylceramide (HexCer).

**Figure 7:**
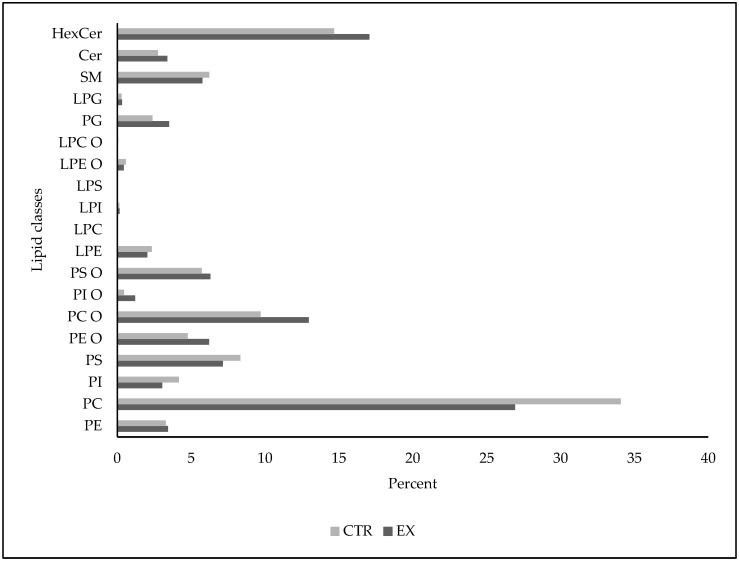
Lipidomic analysis of the rat brain. Animals were sacrificed after 4 months of treatment with SM-VD3, 1 h after the daily dose. The brain was removed, as reported in Section 4, and 1 hemisphere was used for the lipid analysis. A separate lipid pool from 6 CTR animals and 6 Ex animals was used. CTR, control animals; Ex, experimental animals. Variations in phosphatidylethanolamine (PE), phosphatidylcholine (PC), phosphatidylserine (PS), phosphatidyliinositol (PI), ether PE (PE O), ether PC (PC O), ether PI (PI O), ether PS (PS O), lyso PE (LPE), lyso PC (LPC), lyso PI (LPI), lysoPS (LPS), ether lyso PE (LPE O), ether lyso PC (LPC O), phosphatidylglycerol (PG), and lyso PG (LPG).

In the brains of CTR rabbits, when considering individual families, GPLs prevailed in terms of percentage, followed by GPL O, Sphs, PG + LPG, and less abundant families, such as LGPLs and LGPLs O. When considering individual species, PC species were the most abundant, followed by HexCer species and then, in order, by PC O, PS, PS O, PE O, SM, PG, PE, Cer, PI, LPE, PI O, and groups of less abundant species such as LPE O, LPI, LPC, LPS (Figure 7).

After 4 months of treatment, the Ex rabbit animals showed a relatively diversified profile, with a decrease in different GPLs, such as PC, PI, and PS, and the respective GPLs O, such as PC O, PI O, PS O, exhibited the greatest -fold increases. Otherwise, PE and PE O showed different, more modest fold changes, with an increase in PE and a reduction in PE O. The lyso molecules stayed relatively constant. Interestingly, the PG and LPG species that were the least abundant exhibited the greatest fold increases. Moreover, the SM species decreased slightly, and both Cer and HexCer increased (Figure 7).

## 3. Discussion

In this study, we have demonstrated the effect of daily intake for 4 months of a SM-VD3 formulation administered in rabbits in addition to the normal diet. This is the first study on the effect of the SM-VD3 combination in vivo. The in vitro results on embryonic hippocampal cells demonstrated that the SM-VD3 combination, in a specific ratio, had a differentiating effect that was significantly superior to that of SM or VD3 alone. In the experimental model used for this study, the ratio outlined in the patent was maintained.

It is known that SM is absorbed in the middle part of the jejunum thanks to the high amount of alkaline sphingomyelinase (alk-SMase) in the enterocytes [22]. Therefore, in the intestinal lumen, SM is degraded to Cer, which is then converted to sphingosine and absorbed by the enterocytes. In the enterocytes, SM is resynthesized and incorporated into the composition of chylomicrons.

Enterocytes are metabolically very active cells and are able to resynthesize not only SM but also all the degraded PLs in the intestinal lumen to allow for their absorption. In addition, VD3 absorption occurs at the intestinal level, partly by passive diffusion and partly thanks to transporters shared with Chol [23]. Here, we demonstrated that SM-VD3 supplementation in rabbits determines an increase in both total PLs and individual PLs, such as PE, PC, SM, PI + PS in the intestinal mucosa. Polar lipids (glycerophospholipids and sphingolipids) influence the stability of emulsions by enhancing intestinal lipid hydrolysis and promoting faster intestinal lipid absorption [24]. Therefore, it is possible to hypothesize that SM facilitates the absorption of all PLs introduced through the diet. Furthermore, the SM present in the SM-VD3 formulation is extracted from natural products and contains a portion of PC and also of PI and PS, in very small quantities. Furthermore, VD3 promotes the absorption of inorganic phosphorus [25], and this could facilitate the synthesis of PL in enterocytes. Since intestinal PLs were evaluated 1 h after administration of the daily feeding, the observed changes are expected to be related to absorption. However, the possibility that the changes in intestinal mucosal PLs may also be due to changes induced by SM-VD3 administration during the 4 months of treatment cannot be excluded.

Our results indicate that the SM-VD3 formulation does not change the structure of the intestine and liver but changes the blood PL profile. SM reaches the brain and other tissues via specific transporters [26]. Human milk is the richest source of SM, and it is responsible for the promotion of the brain and nervous system [27]. Adding milk fat globules, which are rich in SM, to infant formula promotes cognitive development in infants and children [28]. Dietary SM is shown to regulate multiple neuroenhancing effects in brain disease [29]. Using the same concentration of SM or VD3 as found in breast milk, a significant differentiation of embryonic hippocampal cells is obtained in vitro [10,30]. Our study defines a previously unidentified role of SM and VD3 on the brain of rabbits. We demonstrate that SM-VD3 promotes the GFAP expression. Our attention was focused on GFAP as the primary marker because it is a widely recognized indicator of astrocytic activation, which is one of the main responses to changes in lipid composition of the central nervous system. Increased GFAP expression has been described in several models of neural plasticity, making it a sensitive marker to assess possible changes in glial reactivity. Although other markers, such as vimentin or S100B, may provide complementary information, GFAP was selected for its well-established association with astrocytic response and its relevance in models of lipid alteration. We evaluated GFAP in the white matter and cortex because these brain regions are known to be involved in neural plasticity and glial remodeling in response to changes in lipid composition. White matter, in particular, is rich in myelin and lipid components, and it is therefore plausible that SM supplementation could influence its physiology. The cortex, on the other hand, represents a key region for the integration of cognitive functions and is often the subject of studies on neuronal plasticity and the interaction between lipids and astrocytes. It has long been known that during the development of the nervous system, astrocytes mature and show variations in GFAP expression [31,32]. Most importantly, in the adult nervous system, astrocytes can increase GFAP expression in response to changes in neuronal activity, such as during learning, memory, or recovery from minor stress. Astrocytes are not simply support cells, but actively participate in the regulation of synaptic networks, varying GFAP expression that can increase in response to physiological changes related to synaptic plasticity [31,32,33,34].

In light of these data, we hypothesized that the brain phospholipidome is also changed with SM + VD3 treatment. The results show a reduction in individual PL families such as PE, PC, PS, and PI and an increase in their respective ethers. Ether-PLs are glycerophospholipids with an ether bond in the sn-1 position of their glycerol backbone [35,36]. These include alkyl ether-PLs, if they have no unsaturation next to the ether bond, and alkenyl (or vinyl) ether-PLs, or plasmalogens, which possess a cis double bond next to the ether bond. Ether-PLs are involved in the constitution of membrane rafts and have antioxidant action [35]. Ether-PLs are synthesized in peroxisome [37], which shows several significant interconnections, including the nuclear receptor for VD3 and the phosphatidylcholine cycle [38]. In the brain, ether-PLs are highly abundant and are major components of the myelin structure [36]. Inflammatory stimuli such as LPS and IL-1β reduce ether-PLs in microglia [39]. Our data provide evidence that the SM-VD3 treatment induces a remodeling of the balance between glicero-PLs and ether-Pls, probably playing a protective role on the brain. It has been demonstrated the VD3 protects against neuronal loss via activation of PPARγ, leading to improved neuronal peroxisomal function and neuronal lipid metabolism [40]. In light of these data and of our results, an interplay between SM and VD3 is evident in supporting brain health. Interestingly, we also found an increase in PG. It is possible that these data are the result of the catabolism of LPGs, which are reduced. Similarly, we also found a reduction in SM and, as a result, an increase in the Cer used, in part, for HexCer synthesis. Cer production from SM is considered crucial for the development of the human brain [41]. In accordance, HexCers are essential for the structure, function, and stability of myelin, as well as for axonal growth in neurons [42].

## 4. Materials and Methods

### 4.1. Animals and Treatment

The experiment was conducted at the farm of the Agricultural University of Ti-rana, Faculty of Veterinary Medicine, Albania. The animals were maintained in accordance with Legislative Decree No. 146, implementing Directive 98/58/EC regarding the protection of animals that are kept for farming purposes. The experimental protocol was run with the permission of the Ministry of Agriculture and Rural Development, National Authority for Veterinary and Plant Protection (prot. 824, 18 May 2021) of Albania, and all procedures were performed accordingly. All efforts were made to minimize animal distress and to use only the number of animals necessary to produce reliable results. Moreover, the veterinarian responsible for the farm examined the rabbits daily for health and welfare states.

A total of 12 New Zealand White male rabbits were randomly assigned to two groups according to the dietary treatment, from 35 days (weaning) until 155 days of age (sacrifice). The control group (*n* = 6 animals, CTRL) was fed a commercial feed that included the following ingredients: dehydrated alfalfa meal (43.0%), wheat bran (30.0%), barley (9.5%), sunflower meal (4.6%), rice bran (4.0%), soybean meal (4.0%), calcium carbonate (2.2%), cane molasses (2.0%), soybean oil (0.4%), and salt (0.3%). The other group was fed the same diet, to which a formulation consisting of SM-rich phospholipids + VD3 was added before pelleting, in a patent-protected ratio, at the dose of 0.64 g/kg of rabbit weight (SM-D3 group, *n* = 6), for 4 months. The animals were marked with progressive numbers so as not to make the CTR and Ex samples identifiable to the operators.

### 4.2. Blood Samples

Blood samples were collected at time 0 (basal) before the administration of the feed and at 1, 2, 3, and 4 (at sacrifice) months of treatment to evaluate plasma lipid concentrations. The samples were withdrawn from the marginal ear vein using a butterfly needle of 24G connected to a syringe of 2.5 mL. Blood samples were inserted into tubes containing EDTA, and immediately centrifuged at 3000× *g* for 15 min; furthermore, plasma was stored frozen until it was assayed for lipid levels.

### 4.3. Tissue Samples and Treatment

After 4 months of treatment, the rabbits were sacrificed by electrical stunning followed by bleeding within 10 s. At the slaughterhouse, the gastrointestinal tract was immediately removed from each rabbit, and sections of the ileum and liver were collected. After the head was separated from the trunk, the skull was opened with scissors. Briefly, one blade of the scissors was placed on the lateral canthus of the right eye and the other in the occipital portion of the skull in a horizontal manner, and a first incision was made; the same operation was repeated on the opposite side. Then, a blade was placed on the medial canthus of the right eye and a transverse incision was made on the anterior part of the skull; the same operation was repeated on the opposite side. Then, the sectioned part of the skull was lifted by lifting the occipital part. At this point, the brain was visualized, and then the olfactory lobes were dissected. Next, the brain was raised with a spatula in its ventral position until the optic chiasm was visualized, which was then sectioned. At this point, always with the help of the spatula, the brain was lifted until the medulla oblongata was visible, which was then sectioned, removing the brain from the skull. Each tissue was divided in 3 aliquots, separately inserted in 15 mL sterile tubes and then stored (i) at −80 °C in (ii) 4% neutral phosphate-buffered formaldehyde and (iii) paraformaldehyde until examination. Each sample was analyzed individually. For the brain, the sections were performed on the frontal lobe of the right hemisphere with the rostro-caudal orientation. The paraffin blocks were sectioned into 4 mm-thick sections. All sections were mounted on silan-coated glass slides. Each slide contained a pair of sections at a distance equal to 140 mm, automatically placed. Sections between pairs 7 and 14 were sampled having excluded the first and the last. Then, sections 7, 9, 11, and 13 were used for immunohistochemistry, whereas sections 8, 10, 12, and 14 were used for immunofluorescence analysis.

### 4.4. Immunohistochemistry Analysis

Four µm-thick sections obtained from previously formalin-fixed and paraffin-embedded tissue blocks were placed on positively charged slides for immunohistochemical staining, using the fully automated BOND RX stainer (Leica Biosystems, Nußloch, Germany): firstly, the Bond Dewax solution was used to remove paraffin from sections, then rehydration and immunostaining were performed on the Bond automated system. As regards the subsequent staining, heat-induced antigen retrieval was performed using the ready-to-use citrate-based BondTM Epitope Retrieval Solution 1 (Leica Biosystems, Newcastle Upon Tyne, UK, catalogue no. Catalogue No.: AR9961) at pH 6 for 30′. Afterwards, the sections were incubated for 30′ with the primary antibody against villin (mouse monoclonal, clone CWWB1, dilution 1:150, Leica Biosystems, Newcastle Upon Tyne, UK, Product Code: NCL-L-VILLIN) and with Hep Par1 (mouse-monoclonal, clone OCH1E5, prediluted RTU—Ready to Use—[total protein concentration: ~10 mg/mL], Biocare Medical, Pacheco, CA, USA, catalogue number: PM 166 AA). A 15’ incubation with the primary antibody against GFAP (porcine-monoclonal, clone GA5, prediluted RTU [total protein concentration ~10 mg/mL], Leica Biosystem Newcastle Upon Tyne, UK, Catalog No: PA0026) was carried out. Subsequently, the ready-to-use BondTM Polymer Refine detection system (Leica Biosystems, Newcastle Upon Tyne, UK, Catalog No: DS9800), which is a biotin-free, polymeric horseradish peroxidase (HRP)-linker antibody conjugate system for the detection of tissue-bound IgG and IgM primary antibodies, was used to visualize the complex formed via a brown precipitate and the hematoxylin for nuclear counterstaining. Eventually, the immunostained slides were detected by Leica DM2000 microscopy equipped with a Leica MC120 HD camera. Ten different, randomly chosen, areas at high-power-field (field diameter = 0.55 mm) were analyzed using a semi-quantitative evaluation of the immunostainings, according to the H-Score, as previously reported [43], which was the result of the intensity of the staining (0 = absent, 1 = mild, 2 = moderate, 3 = intense) multiplied by the percentage of labelled cells obtained as the mean of the 10 HPF read.

### 4.5. Immunofluorescence

The sections were incubated over night with 3% (*w*/*v*) BSA, 1% (*w*/*v*) glycine in PBS to block non-specific sites, as previously reported [44]. Then sections were incubated with anti-GFAP antibody diluted 1:100 in 3% (*w*/*v*) BSA in PBS for 1 h, washed three times in 0.1% (*v*/*v*) Tween-20 in PBS and twice in PBS, incubated with tetramethylrhodamineisothiocyanate (TRITC)-conjugated anti-rabbit IgG for 1 h, diluted 1:50 in 3% (*w*/*v*) BSA in PBS and washed as above. The antibody incubations were performed in a humid chamber, at room temperature. Nuclei were counterstained with diamidino-2-phenylindole (DAPI). The samples were mounted in 80% (*w*/*v*) glycerol, containing 0.02% (*w*/*v*) NaN_3_ and *p*-phenylenediamine (1 mg/mL) in PBS to prevent fluorescence fading. Fluorescent analysis was performed on a DMRB Leika epi-fluorescent microscope equipped with a digital camera. 

### 4.6. Lipid Analysis

Total lipids were extracted from the plasma using chloroform/methanol (2:1, *v*/*v*), and the total amount of PLs was evaluated by measuring inorganic phosphorous. Each phospholipid (PL) was separated by thin layer chromatography (TLC) on silica gel using chloroform:methanol:ammonia, 65:25:4 *v*/*v*/*v* and identified with commercial PLs as markers. The PLs were detected using iodine vapors. The spot was scraped into test tubes for the determination of inorganic phosphorous.

### 4.7. LC-MS Analysis

Lipid extraction was performed using the MMC method, as described by Pellegrino et al. [45]. We performed lipidomic analysis on a pool of lipids extracted from the CTR samples and on a pool of lipids extracted from the Ex samples, representing the entire class of Ex versus the entire class of CTR. We chose to perform untargeted lipidomics to obtain a global vision of the lipid species present in the samples. Briefly, 50 µL of homogenate was mixed with 1 mL of a methanol/methyl tert-butyl ether/chloroform (MeOH/MTBE/CHCl_3_) solution in a 1:1:1 ratio. The mixture was vortexed and incubated on ice water for 20 min, followed by agitation in a thermomixer at room temperature (RT) for 20 min at 1900 rpm. After centrifugation at 16,000× *g* for 10 min at 4 °C, the supernatant was transferred to a vial, evaporated to dryness using N_2_, and reconstituted in 400 µL of a methanol/toluene (MeOH/toluene) solution in a 9:1 ratio. The samples were immediately analyzed by LC-MS. Lipid analysis was performed using an Agilent 6530 LC-MS QTOF system (Agilent Technologies, Inc. Headquarters, Santa Clara, CA, USA). Chromatographic separation was carried out on a Waters Acquity CSH column (1.7 µm, 2.1 × 100 mm) maintained at 55 °C. The mobile phase consisted of solvent A (water with 10 mM ammonium acetate), solvent B (acetonitrile), and solvent C (isopropanol with 10 mM ammonium acetate). The flow rate was set at 0.35 mL/min, and the gradient elution program was as follows:
**Time (min)****A (%)****B (%)****C (%)**0.04133262.529.7530.539.754.020.7528.550.7511.011.7526.561.7514.05.4525.169.4520.05.4525.169.4522.05.4825.169.4222.129.7530.539.7525.0Stop Run



Data were acquired in data-dependent acquisition (DDA) mode over a mass range of 40–1700 *m*/*z* in both positive and negative ionization modes. The Agilent JetStream electrospray ionization source was used with the following parameters: capillary voltage (Vcap) set at 4000 V, gas temperature at 300 °C with a flow rate of 12 L/min, nebulizer pressure at 50 psi, sheath gas temperature at 300 °C with a flow rate of 12 L/min, and fragmentor voltage set at 130 V.

### 4.8. Statistical Analyses

The analysis was performed on the 6 control and 6 experimental animals. The data were reported as mean ± SD of 6 samples performed in duplicate. To verify the assumption of normality for the analyzed variables, the Shapiro–Wilk test was applied. The test was performed on all the variables included in this study to confirm the normal distribution of the data and ensure the validity of subsequent statistical analyses. The results of the Shapiro–Wilk test indicated that all the analyzed variables followed a normal distribution (*p* > 0.05), thereby meeting the assumption of normality necessary for the application of parametric tests in subsequent analyses. Therefore, the *t*-test was used. All analyses were performed using SPSS software (version 26).

## 5. Conclusions

In summary, we found very positive effects of SM + VD3 in the brain. In general, the differentiative markers of intestine and liver cells did not change with the treatment, and GFAP increased strongly in the white substance of the brain. This is relevant, considering that both SM and VD3 are essential for brain pathophysiology, and that GFAP expression increases in response to changes in neuronal activity, such as during learning, memory, or recovery from minor stress, and in response to physiological changes related to synaptic plasticity. In addition, we observed a remodeling of the balance between glicero-PLs and ether-Pls, an increase in Cer and HexCer, which are molecules relevant for the structure, function, and stability of myelin. Taken together, these findings provide clues as to how the combination SM + VD3 may play a vital role in normal brain physiology and may ultimately be exploited in the reduction or treatment of neurodegenerative diseases. Our results suggest that the product could be used in the future to facilitate the maturation of the nervous system in premature infants with the aim of preventing neurodegenerative diseases.

## Data Availability

Data is contained within the article (and Supplementary Materials).

## Supplementary Materials

The following supporting information can be downloaded at: https://www.mdpi.com/article/10.3390/ijms26073269/s1.
