# Supplementary material for: Effect of Sphingomyelin and Vitamin D3 Intake on the Rabbit Brain
Source: Int J Mol Sci. 2025 Apr 1;26(7):3269. doi: 10.3390/ijms26073269 (PMC11989450; doi:10.3390/ijms26073269)
Supplement: Supplementary file 1 [file ijms-26-03269-s001.zip › ijms-3504878-supplementary.pdf]

	T0	T1	T2	T3	T4
CTR 1	2,2	2,8	3,55	3,85	4,3
CTR 2	2,35	2,74	3,05	4,12	4,45
CTR 3	2,39	2,95	3,65	3,81	4,05
CTR 4	2,52	2,85	3,38	3,71	4
CTR 5	2,31	2,9	3,59	3,8	4,4
CTR 6	2,28	2,99	3,61	3,85	4,51
Ex 1	2,401	2,55	2,75	3,15	3,75
Ex 2	2,35	2,8	3,2	3,7	4,05
Ex 3	2,65	2,7	3,1	3,45	3,75
Ex 4	2,4	2,67	3,01	3,47	3,94
Ex 5	2,45	2,72	2,8	3,3	4,25
Ex 6	2,493	2,75	3,25	3,8	3,85

Supplemental 1. Weight of rabbits. Data related to Fig.1

	T0	T1	T2	T3	T4
CTR 1	1,231	1,175	1,324	1,315	1,255
CTR 2	1,235	1,171	1,205	1,21	0,959
CTR 3	1,335	1,361	1,22	1,328	0,947
CTR 4	1,31	1,268	1,36	1,26	1,12
CTR 5	1,046	0,916	1,322	1,173	0,965
CTR 6	1,236	1,15	1,354	1,219	1,205
Ex 1	0,942	1,255	1,455	1,299	1,477
Ex 2	1,238	1,438	1,408	1,357	1,553
Ex 3	1,237	1,399	1,277	1,4	1,589
Ex 4	1,281	1,421	1,398	1,269	1,503
Ex 5	0,96	1,511	1,521	1,574	1,441
Ex 6	1,46	1,365	1,315	1,362	1,588

Supplemental 2. Total phospholipid in plasma of rabbits. Data related to Fig.2a

T0				
	PS+PI	SM	PC	PE
CTR 1	209,88	112,11	450,5	99,03
CTR 2	183,9	99,8	493,4	87,4
CTR 3	188	125	464,7	95,2
CTR 4	181,12	102,1	473,6	107,1
CTR 5	155	73,61	474,3	82,17
CTR 6	188,99	92,44	470,42	105,2
Ex 1	210.9	106,22	477,2	130,73
Ex 2	230,55	83,25	499,54	113,14
Ex 3	164,8	73,6	379,2	93,8
Ex 4	210,4	114,9	465,6	108,7
Ex 5	212,7	144,75	484,8	127,9
Ex 6	239,56	168,97	551,84	141,17

T1				
	PS+PI	SM	PC	PE
CTR 1	189,4	131,98	487,7	99,2
CTR 2	166	127,5	515	102,5
CTR 3	180,27	88,86	459,15	112,02
CTR 4	186,8	107,55	463,2	103,2
CTR 5	181,6	103,33	497,58	103,25
CTR 6	214,67	122,88	491,2	118,06
Ex 1	182,48	192,3	651,23	125,7
Ex 2	199,8	144,2	645,4	129,18
Ex 3	206,29	137,7	747,79	122,4
Ex 4	188,06	186,82	598,5	123,03
Ex 5	194,15	157,93	644,13	131,23
Ex 6	201,2	172,33	538	134,53

T2				
	PS+PI	SM	PC	PE
CTR 1	196,44	122,65	489,06	110,72
CTR 2	212,25	43,82	450,5	95,85
CTR 3	161,28	66,69	414,02	103,05
CTR 4	207,54	106,12	469,33	106,71
CTR 5	198,99	105,5	537,06	120,35
CTR 6	192,96	185,73	478,3	83,84
Ex 1	259,29	132,02	705,94	140,4
Ex 2	220,88	152,5	665,03	130,41
Ex 3	177,09	209,03	613,48	139,35
Ex 4	229,4	157,21	657,2	142,08
Ex 5	219,16	125,41	771,52	154,38
Ex 6	186,129	160,17	589,1	139,78

T3				
	PS+PI	SM	PC	PE
CTR 1	168,19	102,75	457,6	94,27
CTR 2	201,69	134,58	481,02	96,73
CTR 3	184,83	113,3	504,02	95,61
CTR 4	172,96	104,56	519	103,73
CTR 5	206,16	58,66	451,44	73,85
CTR 6	175,37	107,89	488,33	101,78
Ex 1	215,62	133,5	583,62	120,7
Ex 2	157,09	154,3	621,3	132,51
Ex 3	202,74	149,63	674,68	160,41
Ex 4	222,7	160,83	617,33	120,92
Ex 5	192,22	139,75	619,14	90,56
Ex 6	174,85	158,9	599,5	93,9

T4				
	PS+PI	SM	PC	PE
CTR 1	183,44	99,21	477,29	110,61
CTR 2	182, 51	113,99	528,28	125,63
CTR 3	175,75	97	407,86	91,61
CTR 4	168,42	96,52	478,64	71,7
CTR 5	204,32	82	512,81	54,67
CTR 6	181,89	93,59	422,5	89,5

T4				
	PS+PI	SM	PC	PE
Ex 1	157,66	129,5	630	90,3
Ex 2	168,77	136,37	561,5	98,1
Ex 3	204,17	133,87	477,3	127,05
Ex 4	166,02	163,88	621,06	112,03
Ex 5	173,83	197,75	658,33	102,08
Ex 6	202,57	190,8	616,95	134,15

Supplemental 3. Each phospholipid in plasma of rabbits. Data related to Fig.2b

	Total PLs	PS	PI	SM	PC	PE
CTR 1	84,47	12,3	16,29	23,72	24,47	8,7
CTR 2	86,8	17,09	15,84	18,33	20,06	11,2
CTR 3	88,43	11,71	16,47	23,77	16,08	12,11
CTR 4	89,21	14,07	16,92	18,35	20,22	9,31
CTR 5	88,89	15,04	17,56	21,02	19,8	9,88
CTR 6	83,75	13,99	16,33	22,8	23,5	10,44
Ex 1	126,4	17,75	35,21	30,7	51,45	12,06
Ex 2	135,5	22,61	33,59	26,84	39,75	15,24
Ex 3	130,1	19,87	30,77	34,42	50,83	13,07
Ex 4	135,8	19,86	38,6	27,93	40,55	10,5
Ex 5	121,7	18,44	34,55	35,4	52,65	10,02
Ex 6	120,7	20,3	32,08	30,4	42,07	12,6

Supplemental 4. Total and each phospholipid in intestine of rabbits. Data related to Fig.3a and b

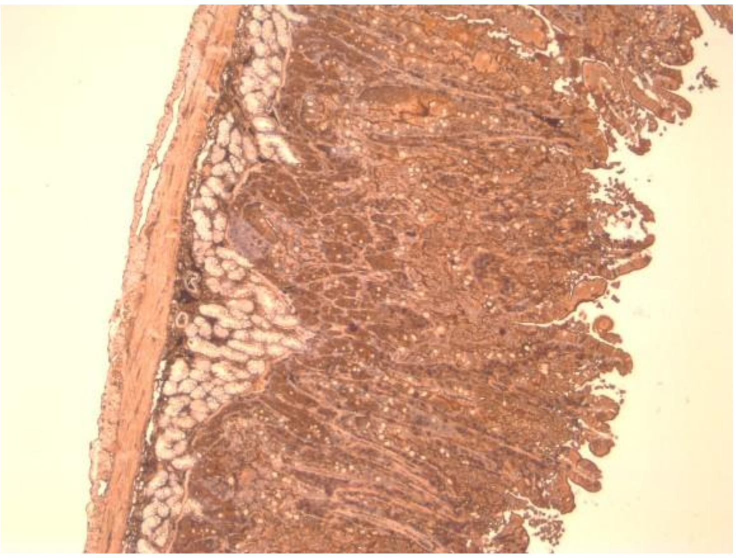

Figure 4a CTR
